# Molecular Prevalence and Phylogenetic Analysis of *Theileria ovis* and *Theileria lestoquardi* in Small Ruminants in Siirt Province, Türkiye

**DOI:** 10.1002/vms3.70522

**Published:** 2025-07-24

**Authors:** Burcak Aslan Celik, Murat Kara, Adnan Ayan, Muhammed Ahmed Selcuk, Ozgur Yasar Celik

**Affiliations:** ^1^ Department of Parasitology Faculty of Veterinary Medicine Siirt University Siirt Turkey; ^2^ Department of Parasitology Faculty of Veterinary Medicine Aksaray University Aksaray Turkey; ^3^ Department of Internal Medicine Faculty of Veterinary Medicine Siirt University Siirt Turkey

**Keywords:** goat, PCR, sheep, Siirt, *Theileria lestoquardi*, *Theileria ovis*

## Abstract

**Background:**

Theileriosis is a haemoparasitic disease of domestic and wild ruminants, caused by *Theileria* species, transmitted by Ixodid tick species, particularly prevalent in tropical and subtropical regions. This study aimed to investigate the prevalence of *Theileria ovis* and *Theileria lestoquardi* in sheep and goats in Siirt province using molecular methods and to analyse their evolutionary relationships with published sequences.

**Materials and Methods:**

The animal material of the study consisted of 350 sheep and 350 goats in Siirt province and its districts. Blood samples were collected from the jugular veins of the animals and put into EDTA tubes. DNA extraction, PCR amplification and sequence analyses were performed on the collected samples.

**Results:**

According to the analysis results, the prevalence of *T. ovis* was found to be 5.43% (19/350, 95% confidence interval [CI] = 3.50%–8.32%) in sheep, 4.57% (16/350, 95% CI = 2.83%–7.30%) in goats and 5% (35/700) in total. A higher prevalence was determined in females (5.20%) and in the 2–3 age group (5.52%). Among sheep breeds, the highest prevalence was detected in Hamdani sheep (8.26%), while in goats, it was detected in Aleppo goats (7.27%). Among locations, the highest prevalence was determined in the Tillo district (10.34%). As a result of the research, *Theileria lestoquardi* was not found in either sheep or goats.

**Conclusion:**

This research confirmed the presence of *T. ovis* in sheep and goats in Siirt province, but *T. lestoquardi* was not detected. In future studies, a more detailed investigation of the presence of vector ticks and the development of regional control strategies is recommended.

## Graphical abstract

According to the analysis results, the prevalence of *T. ovis* was found to be 5.43% in sheep, 4.57% in goats and 5% in total. *Theileria lestoquardi* was not found in either sheep or goats.

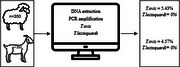

## 1 Introduction

Sheep and goats can be infected with many pathogens, including a wide variety of parasites. Among the common haemoparasites in ruminants are protozoan parasites such as *Theileria*, *Babesia* and *Trypanosoma* (Almahallawi et al. 2024).

Theileriosis is an important haemoparasitic disease transmitted by vector ticks, caused by protozoan parasites belonging to the genus *Theileria* (phylum Apicomplexa, order Piroplasmida, family Theileriidae, subclass Piroplasmia), which infect various domestic and wild animals in different parts of the world and cause significant economic losses in livestock, especially in tropical and subtropical regions (Altay et al. 2004; Li et al. 2014; Kho et al. 2017; Luo et al. 2017; Mohammadi et al. 2017; Islam et al. 2021).

The genus *Theileria* includes more than 185 different species, and pathogenic species predominantly cause disease in domestic ruminants and horses (Al‐Fahdi et al. 2017). Small ruminant theileriosis can be caused by at least six *Theileria* species, including *Theileria ovis, Theileria lestoquardi, Theileria luwenshuni, Theileria uilenbergi, Theileria separata, Theileria annulata* and *Theileria recondita* (Aziz and Hamadamin 2025). Among these species, *T. lestoquardi* (*Theileria hirci*), *T. luwenshuni* and *T. uilenbergi* are considered highly pathogenic, while *T. ovis*, *T. recondita* and *T. separata* are among the non‐pathogenic or mildly pathogenic *Theileria* species in small ruminants (Aktaş, Altay, et al. 2005; Li et al. 2014; Mohammadi et al. 2017; Almahallawi et al. 2024). These pathogens are transmitted by ticks belonging to the Ixodidae family, including species such as *Hyalomma* spp., *Haemaphysalis*, *Dermacentor*, *Rhipicephalus* and *Amblyomma* (Aktaş et al. 2006; Zaeemi et al. 2011; Alanazi et al. 2019; Aziz and Hamadamin 2025).

*Theileria* species cause clinical and subclinical infections in many wild and domestic animals, including sheep and goats (Luo et al. 2017).

Characteristic clinical symptoms include fever, anaemia, enlargement of superficial lymph nodes, decreased appetite, respiratory distress and general weakness (Ge et al. 2012; Mohammadi et al. 2017; Alanazi et al. 2019; Aziz and Hamadamin 2025).

This study aimed to investigate the prevalence of *T. ovis* and *T. lestoquardi* in sheep and goats in Siirt province, located in the Southeastern Anatolia region of Türkiye, using molecular methods and to analyse their evolutionary relationships with published sequences.

## 2 Materials and Methods

### 2.1 Study Area and Sample Collection

**FIGURE 1:**
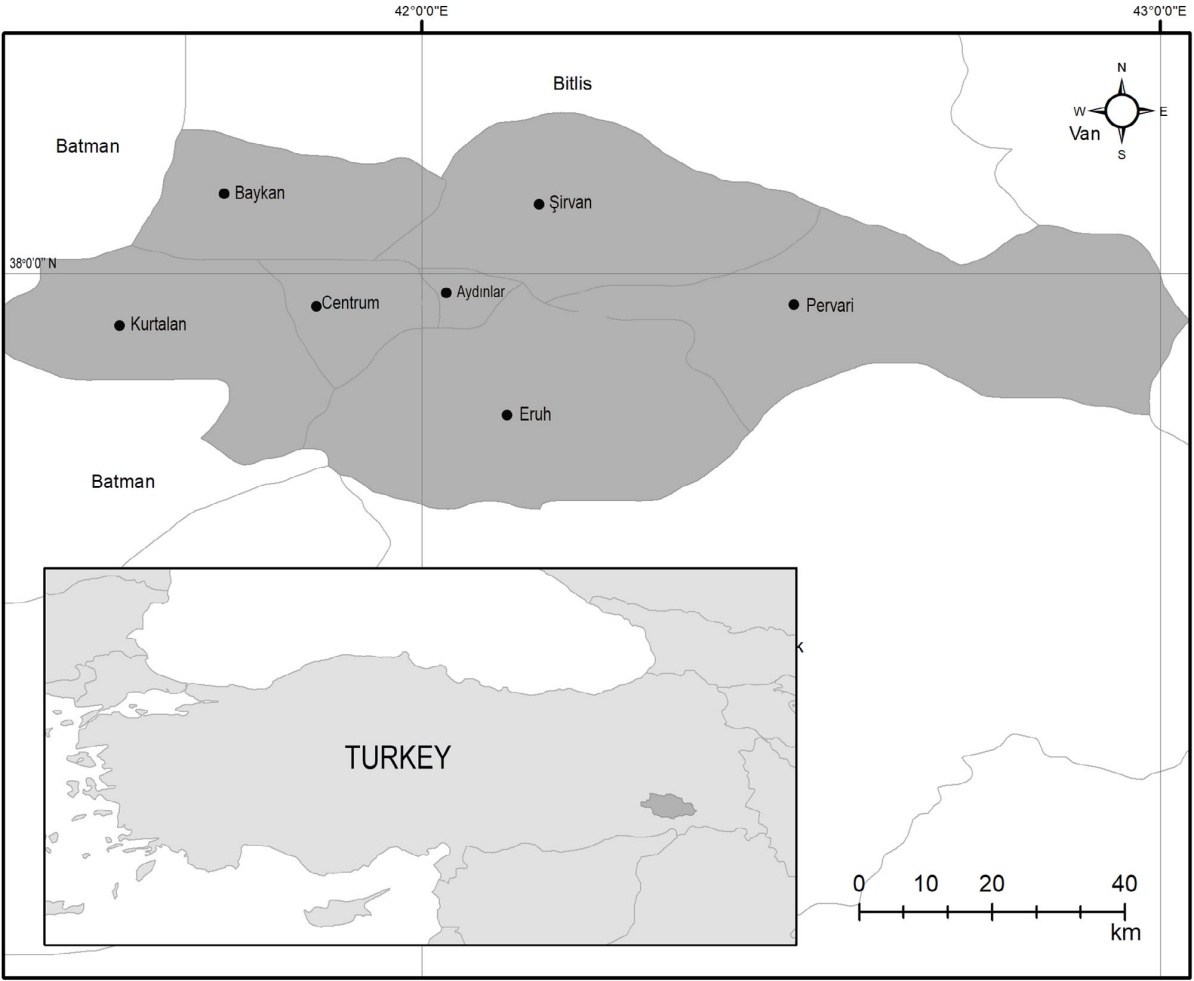
Map of Siirt province where study samples were collected.

In this study, blood samples were collected from a total of 700 small ruminants (350 sheep and 350 goats) randomly selected from herds located in Siirt province and its districts between May 2024 and October 2024, which corresponds to the active transmission season for vector‐borne infections. Sampling was conducted across seven different locations: Centre, Eruh, Kurtalan, Aydınlar, Baykan, Şirvan and Pervari (Figure 1). Blood samples were collected from sheep and goats via the jugular vein into EDTA‐containing tubes. Information regarding the location, breed, age, sex and tick infestation status of each animal was recorded. The collected samples were stored at −20°C at the Department of Parasitology, Faculty of Veterinary Medicine, Siirt University, until further analysis.

### 2.2 DNA Extraction

Genomic DNA (gDNA) isolation from blood samples was performed using a commercial kit, ABT DNA Isolation Kit (ABT Laboratory Industry, Ankara, Türkiye). After completion of gDNA isolation, the A260/A280 ratios of gDNA were measured using Nanodrop (Qubit Fluorometric Quantitation System, USA). After the measurement procedures were completed, DNA samples were stored at −20°C for use in molecular analyses.

### 2.3 PCR Amplification

Nested PCR analysis was performed on the isolated gDNA samples. In the first round of amplification, the forward primer Nbab‐1F (5′‐AAGCCATGCATGTCTAAGTATAAGCTTTT‐3′) and the reverse primer Nbab‐1R (5′‐CTTCTCCTTCCTTTAAGTGATAAGGTTCAC‐3′) were used to amplify approximately 1600 bp of the 18S rRNA gene region targeting *Theileria* spp. (Oosthuizen et al. 2008). In the second round of amplification, species‐specific internal primers were used to achieve more sensitive amplification of the target regions. For *T. ovis*, the target region of approximately 520 bp was amplified using the forward primer TSsr 170F (5′‐TCGAGACCTTCGGGT‐3′) and the reverse primer TSsr 670R (5′‐AAAGACTCGTAAAGGAGCAA‐3′). For *T. lestoquardi*, the target region of approximately 785 bp was amplified using the forward primer TL‐F (5′‐GTGCCGCAAGTGAGTCA‐3′) and the reverse primer TL‐R (5′‐GGACTGATGAGAAGACGATGAG‐3′) (Kirvar et al. 1998; Aktaş et al. 2006). The PCR reaction mixture was prepared to a final volume of 25 µL, containing 10 µL of A.B.T. 2X PCR Master Mix (ABT Laboratory Industry, Ankara, Türkiye), 1 µL (20 pmol/µL) of each outer and inner primer, 2.5 µL of template DNA and 10.5 µL of ddH₂O. The thermal cycling profiles were applied according to the protocols described by Oosthuizen et al. (2008) for *Theileria* spp., Aktaş et al. (2006) for *T. ovis* and Kirvar et al. (1998) for *T. lestoquardi*. PCR products were electrophoresed on a 1.5% agarose gel at 90 V for approximately 60 min and visualized under UV light using a gel documentation system (GEN‐BOX imagER FX, Türkiye).

### 2.4 Sequence Analysis and Phylogenetic Evaluation

A total of six samples (four sheep and two goats) suitable for sequencing were selected from among the PCR‐positive samples showing the most prominent bands and sent to a private company (ABT Laboratory Industry, Ankara, Türkiye) for DNA sequence analysis. The obtained DNA sequences were checked, aligned and made suitable for analysis using MAFFT Version 7 software. The edited sequences were submitted to the NCBI database for BLASTn search, sequence alignment and analysis (Altschul et al. 1990). In the phylogenetic tree construction process, sequences were aligned with MAFFT Version 7 software, then a model test was performed using the Maximum Likelihood (ML) statistical method in MEGA X software. According to the Bayesian Information Criterion (BIC) analysis, the K2+G+I model was determined to be the most suitable, and a phylogenetic tree was constructed with 1000 bootstrap replications according to this model (Kumar et al. 2018).

### 2.5 Statistical Analyses

The data obtained in the study were analysed using SPSS V16.0 (IBM, Chicago, IL, USA) software. The relationship between grouped variables was calculated using the chi‐square test. A difference was considered statistically significant when *p* < 0.05.

## 3 Results

**FIGURE 2:**
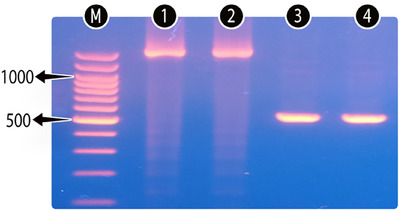
Appearance of bands formed as a result of PCR amplification of the 18S ribosomal ribonucleic acid (18S rRNA) gene. M: DNA marker (100 bp), 1–2: Region amplified with NBAB primers (1600 bp), 3–4: *Theileria ovis* (520 bp).

As a result of the analyses, *T. lestoquardi* was not found in either sheep or goats, while the total prevalence of *T. ovis* was determined to be 5% (35/700) (Figure 2). This prevalence was determined as 5.43% (19/350, 95% confidence interval [CI] = 3.50%–8.32%) in sheep and 4.57% (16/350, 95% CI = 2.83%–7.30%) in goats (*p* > 0.05). Among genders, positivity was detected in 5.20% (30/577) of females and 4.07% (5/123) of males (*p* > 0.05). When looking at age groups, prevalence was determined as 3.01% (4/133) in those one year old and under, 5.52% (19/344) in the 2–3 age group and 5.38% (12/223) in those 4 years old and over (*p* > 0.05). Looking at sheep breeds, positivity rates of 8.26%, 1.03% and 0% were detected among Hamdani, Romanov and Assaf breeds, respectively (*p* < 0.05). Looking at goat breeds, positivity rates of 7.27%, 3.70% and 0% were detected among Aleppo, Hair and Saanen goats, respectively (*p* > 0.05). Among locations, the highest prevalence was detected in Tillo district (10.34%), and the lowest prevalence was detected in Baykan district (2.25%) (*p* > 0.05) (Table 1).

**TABLE 1:** Distribution of *Theileria ovis* and *Theileria lestoquardi* prevalence according to species, breed, sex, age and location.

Parameters	Examined	*T. ovis*		*T. lestoquardi*		*p*
	(*n*)	*n*	(%)	*n*	(%)	
Breed						
Sheep	350	19	5.43	0	0.00	0.60
Goats	350	16	4.57	0	0.00	
Sheep						
Hamdani	218	18	8.26	0	0.00	0.01
Romanov	97	1	1.03	0	0.00	
Assaf	35	0	0.00	0	0.00	
Goat						
Hair	216	8	3.70	0	0.00	0.19
Aleppo	110	8	7.27	0	0.00	
Saanen	24	0	0.00	0	0.00	
Sex						
Female	577	30	5.20	0	0.00	0.60
Male	123	5	4.07	0	0.00	
Age (Year)						
≤ 1	133	4	3.01	0	0.00	0.83
2–3	344	19	5.52	0	0.00	
≥ 4	223	12	5.38	0	0.00	
Location						
Eruh	80	3	3.75	0	0.00	0.43
Centre	360	17	4.72	0	0.00	
Kurtalan	53	5	9.43	0	0.00	
Tillo	29	3	10.34	0	0.00	
Baykan	89	2	2.25	0	0.00	
Şirvan	61	3	4.92	0	0.00	
Pervari	28	2	7.14	0	0.00	
Overall	**700**	**35**	**5.00**	**0**	**0.00**	

In this study, small ruminants were examined for the presence of ectoparasites, and tick samples were collected from a total of 123 goats and 83 sheep. Morphological identification of all collected tick specimens revealed that they belonged exclusively to the species *Rhipicephalus bursa*.

**FIGURE 3:**
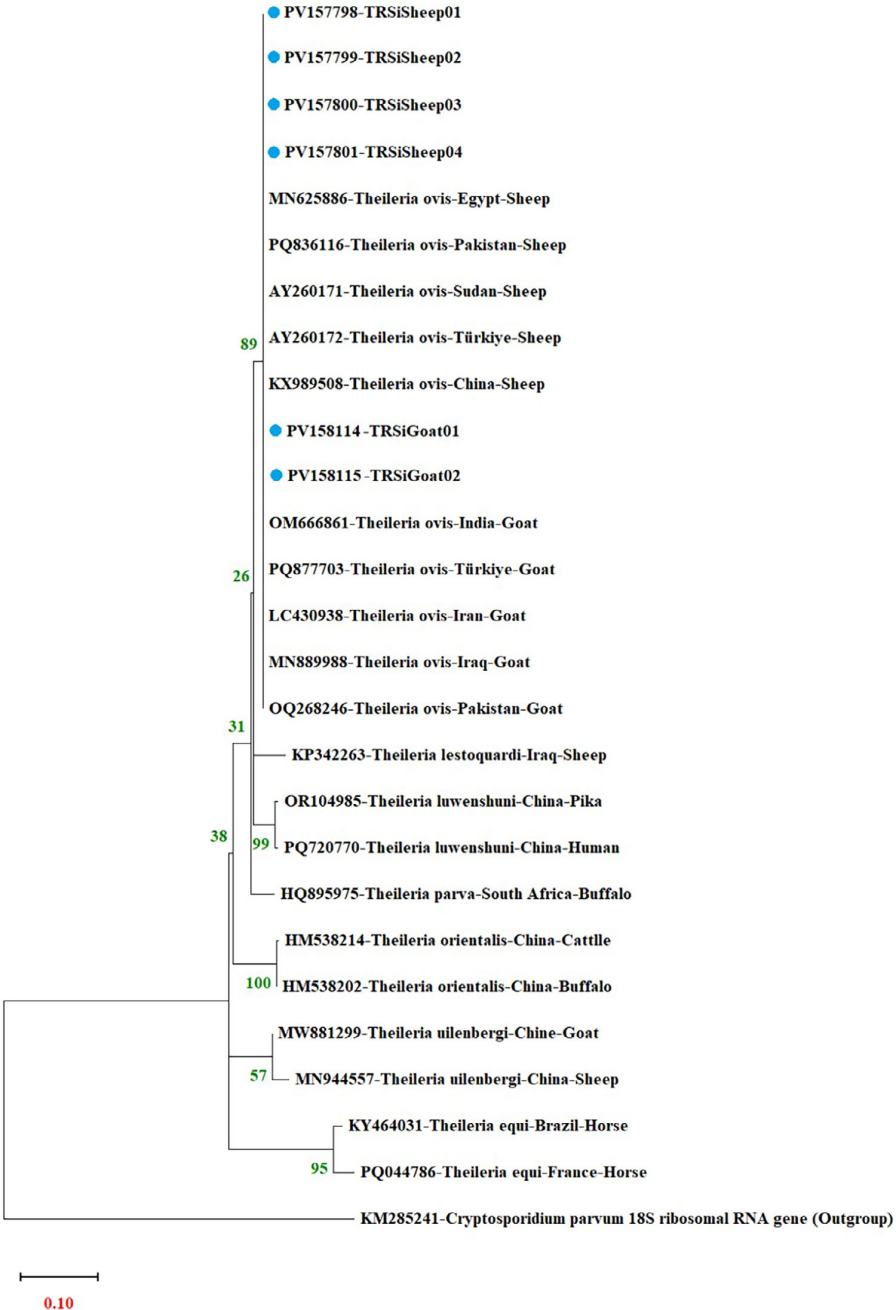
Phylogenetic tree view constructed with 18S rRNA gene region sequences (424 bp) of *T. ovis*. The phylogenetic tree was constructed with MEGA X version using Kimura 2‐Parameter + Gamma Distribution + Invariant Sites (K2+G+I).

In this study, six samples were selected for sequencing from a total of 35 PCR‐positive samples, and the obtained sequences were submitted to the GenBank database. During the selection of samples, priority was given to those demonstrating high amplification quality, with additional consideration to represent diverse geographic regions and host species. This approach aimed to achieve a representative selection that ensured technical reliability and reflected epidemiological diversity. The obtained sequences were compared with reference sequences in GenBank using the NCBI Basic Local Alignment Search Tool (BLASTn), revealing a 100% similarity to *T. ovis* for all samples. These sequences were aligned and edited based on reference sequences and subsequently submitted to GenBank, with accession numbers provided in Table 2. The phylogenetic relationships of these sequenced samples with other *Theileria* species recorded in international databases were evaluated using a constructed phylogenetic tree (Figure 3).

**TABLE 2:** Comparison of results with the database in NCBI Basic Local Alignment Search Tool.

Pathogen	Host	Target gen	Accession no	Length
*T. ovis*	Sheep	18s rRNA	PV157798	424 bp
*T. ovis*	Sheep	18s rRNA	PV157799	424 bp
*T. ovis*	Sheep	18s rRNA	PV157800	424 bp
*T. ovis*	Sheep	18s rRNA	PV157801	424 bp
*T. ovis*	Goat	18s rRNA	PV158114	424 bp
*T. ovis*	Goat	18s rRNA	PV158115	424 bp

The phylogenetic relationships of molecularly characterized *T. ovis* isolates with different species reported from various parts of the world are shown in the phylogenetic tree. The phylogenetic resolution at the species level was strongly supported by high bootstrap values in the ML analyses. For these analyses, highly similar reference sequences of the 18S rRNA gene region (424 bp) identified via BLAST [MN625886, P836116, AY260171, AY260172, KX989508, OM666861, P877703, LC430938, MN889988, OQ268246], along with other species obtained from the NCBI‐PubMed database (*T. lestoquardi* [KP342263], *T. luwenshuni* [OR104985, PQ720770], *T. parva* [HQ895975], *T. orientalis* [HM538214, HM538202], *T. uilenbergi* [MW881299, MN944557], *T. equi* [KY464031, PQ044786]), were utilized. *Cryptosporidium parvum* (KM285241) was selected as an outgroup. These sequences served as references for accurate species identification and phylogenetic assessment.

Phylogenetic analyses demonstrated that Turkish *T. ovis* isolates from both sheep and goats exhibited high genetic similarity and clustered within a monophyletic clade. Turkish isolates grouped closely with other geographically diverse *T. ovis* isolates from various countries, forming a strongly supported cluster with a bootstrap value of 89. This indicates that *T. ovis* maintains a highly conserved genetic structure despite its wide geographical distribution. Conversely, other *Theileria* species were clearly differentiated phylogenetically, each forming homogeneous and genetically distinct clades supported by high bootstrap values.

## 4 Discussion

Vector‐borne diseases threaten both human and animal health. These diseases also cause significant economic losses due to high mortality rates and reduced productivity in livestock (Radwan and El Kelesh 2009). Species causing theileriosis in sheep and goats are reported to cause clinical and subclinical infections in tropical and subtropical regions (Aktaş, Dumanli, et al. 2005; Mohammadi et al. 2017).

Many studies have been conducted in different countries to determine the prevalence of *Theileria* in small ruminants worldwide. In studies conducted, prevalence rates were reported as 40% in sheep and 34.7% in goats (*T. ovis*) in Saudi Arabia (Alanazi et al. 2019), 8.50% in goats (*Theileria* spp.) in Bangladesh (Islam et al. 2021), 26% in sheep (*Theileria* spp.) in China (Ge et al. 2012), 21.77% in goats (Luo et al. 2017), 93.8% in sheep and 1.9% in goats (*Theileria* spp.) in Ethiopia (Gebrekidan et al. 2014), 32% in goats (*Theileria* spp.) in India (Arif et al. 2025), 40.2% in sheep (*T. ovis*) in Iran (Zaeemi et al. 2011), 17% in goats (*T. ovis*: 85.3%, *T. lestoquardi*: 11.7%) (Rahmani‐Varmale et al. 2019), 47.7% in sheep and 38.3% in goats (*Theileria* spp.) in Iraq (Aziz and Hamadamin 2025), 63.4% in sheep (*Theileria* spp.) in Spain (Nagore et al. 2004), 21.33% in sheep (*T. ovis*) in Egypt (Radwan and El Kelesh 2009), 28% in goats (*Theileria* spp.) (Radwan and El Kelesh 2009), 36.7% in sheep (*T. ovis*: 14.5%, *T. lestoquardi*: 3.0%) and 2.7% in goats (*T. ovis*: 1.5%) in Oman (Al‐Fahdi et al. 2017) and 13.92% in sheep and 8.20% in goats (*Theileria* spp.) in Pakistan (Naz et al. 2012). In studies conducted in different regions of Türkiye, 18.40%–79.69% prevalence was reported in sheep and 0.33%–11.27% in goats (Inci et al. 2003; Altay et al. 2004; Aktaş, Dumanli, et al. 2005; M. F. Aydın et al. 2013; Altay et al. 2017; N. Aydın et al. 2022; Kose et al. 2022).

Although different diagnostic methods are available for the detection of the disease, molecular methods are reported to have higher efficacy (Radwan and El Kelesh 2009; Rahmani‐Varmale et al. 2019). In this study, molecular methods were also used, and the prevalence of *T. ovis* was found to be 5.43% in sheep and 4.57% in goats. While these results are similar to the findings of some researchers (Al‐Fahdi et al. 2017; Mohammadi et al. 2017; Islam et al. 2021), they were found to be lower than the results of some studies (Nagore et al. 2004; Radwan and El Kelesh 2009; Zaeemi et al. 2011; Ge et al. 2012; Gebrekidan et al. 2014; Kho et al. 2017; Alanazi et al. 2019; Rahmani‐Varmale et al. 2019; Arif et al. 2025; Aziz and Hamadamin 2025).

In studies conducted to determine the prevalence of *T. lestoquardi*, prevalence rates of 2.31% in Egypt (Radwan and El Kelesh 2009), 3% in Pakistan (Saeed et al. 2015) and 6.25% in Iran (Mohammadi et al. 2017) have been reported. In this study, the presence of T. lestoquardi was not detected in either sheep or goats. This situation may be due to the absence or lack of infectious ticks in the environment or infectious ticks not acting as vectors to transmit *Theileria*.

In studies conducted, some researchers (Durrani et al. 2012; Naz et al. 2012; Gebrekidan et al. 2014; Luo et al. 2017; Mohammadi et al. 2017; Alanazi et al. 2019; Arif et al. 2025) reported higher prevalence in females, while some researchers (Naz et al. 2012; Alanazi et al. 2019; Hassan et al. 2019; Rahmani‐Varmale et al. 2019; Riaz et al. 2019; Islam et al. 2021; Almahallawi et al. 2024; Aziz and Hamadamin 2025) reported higher prevalence in males. In this study, a higher prevalence was detected in females (5.20%) compared to males (4.07%), but no statistically significant difference was found (*p* > 0.05). The higher prevalence observed in females may be due to immunosuppression that occurs particularly during pregnancy, parturition and lactation. Additionally, in herds, female animals are generally kept as breeding stock for longer periods compared to males. This increases their duration of exposure to ticks and infectious agents. In contrast, male animals are usually sent for slaughter at a younger age, which may result in shorter exposure times.

Studies conducted worldwide have generally reported a higher prevalence in individuals over 1 year of age (Aktaş, Dumanli, et al. 2005; Durrani et al. 2012; Alanazi et al. 2019; Hassan et al. 2019; Riaz et al. 2019; Aziz and Hamadamin 2025). In the present study, the lowest prevalence was observed in animals aged one year and under, while the highest prevalence was detected in the 2–3‐year age group. However, the difference between age groups was not statistically significant (*p* > 0.05). These findings are consistent with the results reported by other researchers (Aktaş, Dumanli, et al. 2005; Alanazi et al. 2019; Rahmani‐Varmale et al. 2019; Riaz et al. 2019; Islam et al. 2021; Aziz and Hamadamin 2025). The higher prevalence of *T. ovis* observed in the 2–3‐year age group may be attributed to several biological and management‐related factors. At this age, animals are typically at their peak of productivity and are more frequently exposed to pasture environments where tick vectors are prevalent. Increased grazing activity during this stage results in greater contact with infected ticks, thereby raising the risk of infection. Additionally, the passive immunity acquired from colostrum in early life has usually waned by this age. Furthermore, 2–3‐year‐old animals may not yet have developed full adaptive immunity due to limited previous exposure to the pathogen, making them more susceptible to primary infections.

In a study conducted in Bangladesh, the prevalence rates among goat breeds were reported as 11.73% in Crossbred, 5.56% in Jamunapari and 5.30% in Black Bengal goats, with Crossbred animals found to be more susceptible (Islam et al. 2021). In a study from Pakistan by Riaz et al. (2019), the prevalence among Nacchi, Beetal and Teddy breeds was reported as 16.6%, 15.8% and 15%, respectively. Another study conducted in Pakistan reported prevalence rates of 17.5% in Beetal, 14.45% in Kajli, 14.21% in Damani, 13.33% in Dera Din Panah, 12.26% in Teddy and 11.42% in Barbari goats (Ullah et al. 2018). In the present study, when sheep breeds were examined, positivity rates were found to be 8.26% in Hamdani, 1.03% in Romanov and 0% in Assaf sheep, with the differences being statistically significant (*p* < 0.05). Regarding goat breeds, the positivity rates were 7.27% in Aleppo goats, 3.70% in Hair goats and 0% in Saanen goats, although the differences were not statistically significant (*p *> 0.05).

The reasons for the differences observed among studies may include geographical conditions, climatic conditions, methods used, prevalence of vector ticks, sampling time and sample size.

In this study, the partial 18S rRNA gene sequences of *T. ovis* obtained from sheep and goats were found to exhibit a high degree of similarity (100%) with isolates from Türkiye (KT851424‐KT851425), Iraq (MN889988–MN889989) and Iran (GU726903‐GU726904) (Zaeemi et al. 2011; Orkun et al. 2016; Hassen and Meerkhan 2020). This high level of genetic similarity provides important insights into the regional dissemination dynamics of the parasite. It is believed that this similarity may be attributed to factors such as geographical proximity, transboundary livestock movements, shared grazing lands and commercial animal trade. The practice of raising animals under free‐grazing systems and the uncontrolled livestock trade with neighbouring countries may accelerate the spread of the parasite, thereby contributing to the observed genetic similarity.

## 5 Conclusion

This study confirmed the presence of *T. ovis* in sheep and goats in Siirt Province; however, *T. lestoquardi* was not detected. The low prevalence rates observed in the region may be associated with the distribution of vector ticks and environmental factors. Future studies are recommended to investigate the presence of vector ticks in greater detail and to develop regional control strategies.

## Author Contributions

**Burçak Aslan Çelik**: conceptualization, writing – original draft, methodology, writing – review and editing. **Murat Kara**: writing – review and editing, methodology. **Adnan Ayan**: methodology, data curation. **Muhammed Ahmed Selçuk**: investigation. **Özgür Yaşar Çelik**: writing – review and editing, investigation.

## Ethics Statement

Ethical approval for this study was obtained from Siirt University Experimental Animals Local Ethics Committee (Decision no.: 2024/01/02).

## Conflicts of Interest

The authors declare no conflicts of interest.

## Peer Review

The peer review history for this article is available at https://www.webofscience.com/api/gateway/wos/peer‐review/10.1002/vms3.70522.

## Data Availability

The data used in this study are available from the corresponding author on reasonable request.
